# Simple recycling of biowaste eggshells to various calcium phosphates for specific industries

**DOI:** 10.1038/s41598-021-94643-1

**Published:** 2021-07-26

**Authors:** Nongnuch Laohavisuti, Banjong Boonchom, Wimonmat Boonmee, Kittichai Chaiseeda, Somkiat Seesanong

**Affiliations:** 1 Department of Animal Production Technology and Fishery, School of Agricultural Technology, King Mongkut’s Institute of Technology Ladkrabang, Bangkok, 10520 Thailand; 2 Advanced Functional Phosphate Material Research Unit, Department of Chemistry, School of Science, King Mongkut’s Institute of Technology Ladkrabang, Bangkok, 10520 Thailand; 3 Municipal Waste and Wastewater Management Learning Center, School of Science, King Mongkut’s Institute of Technology Ladkrabang, Bangkok, 10520 Thailand; 4 Department of Biology, School of Science, King Mongkut’s Institute of Technology Ladkrabang, Bangkok, 10520 Thailand; 5 Organic Synthesis, Electrochemistry and Natural Product Research Unit (OSEN), Department of Chemistry, Faculty of Science, King Mongkut’s University of Technology Thonburi, Bangkok, 10140 Thailand; 6 Department of Plant Production Technology, School of Agricultural Technology, King Mongkut’s Institute of Technology Ladkrabang, Bangkok, 10520 Thailand

**Keywords:** Environmental sciences, Chemistry, Materials science

## Abstract

Egg consumption is very high throughout the world and with it comes enormous amount of waste eggshells. To reduce and utilize these wastes, eggshell wastes were simply transformed to low- or high-purity calcium carbonate grades by washing, crushing, and drying to use as raw materials for producing highly valuable calcium phosphate products. Low-purity calcium carbonate grade was used to prepare triple superphosphate for using in fertilizer industry, whereas high-purity calcium carbonate grade was used to produce dicalcium phosphate dihydrate, monocalcium phosphate monohydrate, and tricalcium phosphate for using in mineral feed and food additive industries. All calcium phosphate samples obtained by simple, rapid, cheap, and environmentally safe method using eggshells and phosphoric acid were identified and their structural phases and impurities were determined by XRF, XRD and FTIR techniques. Thermal behaviors of raw materials and the prepared calcium phosphates excepted tricalcium phosphate were investigated by TG/DTG techniques. The methodologies described here will be useful to manage eggshells by converting them to highly valuable products, which can solve eggshell wastes problem from industries and communities. This finding supports the viewpoint of zero waste operation to produce value-added products for obtaining sustainable development, which may be selected as an alternative way for material recycling and waste management in the future.

Agro-food from fields of industries and communities produce a substantial amount of pollution every day, so it is becoming urgent to manage this problem^1^. As the restriction connected to environmental issues is becoming quite rigorous, it is necessary to search and develop treating systems for agro-food wastes^1–6^. Globally, approximately 83 million tons of eggs were produced in 2018, which of course would produce a lot of waste eggshells^7^. As food, eggs (chicken, duck, ostrich, turkeys, goose, and quail eggs) are used in many food menus for consumption each day and egg products in the industrialized food production (e.g., powder, liquid, and frozen forms) offer economic benefits^1^. In the case of agro industry, the farming of different types of animal species has a number of waste eggshells after eggs are hatched. In Thailand, egg consumption has used significant amounts of egg that account for more than 15,000 million eggs a year^8^. From previous research, average weight of an egg is about 60 g, generating shell about 11%; thus, the produced wastes can be estimated as being around 90,000 tons per year^1^. Every day thousands of eggshells are being discarded, causing serious environmental problem including unpleasant smell, noise of insects and abrasiveness of the eggshells. These wastes could be recycled and converted to valuable calcium compounds by different methods in an environmentally sound nature, which is a good choice for zero waste management.

Fundamentally, eggshells are composed of a network of protein fibers, connected with crystals of calcium carbonate (> 96% of shell weight), magnesium carbonate (< 1%), calcium phosphate (< 1%), magnesium phosphate (< 1%), silicon oxide (< 1%), and also of organic matters and water. The major composition of the shell, calcium carbonate (CaCO_3_), is a crystal that occurs in the natural form of calcite phase^1^. Therefore, the use of these eggshells, collected greatly every day, as an alternative source of CaCO_3_, will help reduce the risk of microbiological and toxic problems, and may reduce the impact on the natural reserves of limestone, non-renewable natural sources. There are several extensive reviews of the applications of eggshells^9–13^. Several ways have been reported to manage eggshells waste typically destined for landfill and some others such as soil conditioner, fertilizer, animal feed, additive food, adsorbent, calcium supplement, paper manufacture, and material re-used through recycling, all having the potential to be more sustainable methods^3,14,15^. Recently, researches have studied on using eggshells for removing dyes^16,17^ and heavy metals^18–24^ from wastewater, adsorbing of phosphorus or phosphate^25,26^, recycling of polyvinyl chloride^27^, synthesis of PbS/CaCO_3_ nanocomposites for degradation of tetracycline^28^, and preparing bio-adsorbent^29^ and composites as catalyst for some reactions^30,31^. Eggshell membrane has also been used to produce biochar-supported Cu^2+^/Cu^+^ composite for ractopamine detection^32^, CuO–ZnO nanocomposites for water purification^33^, and nano-silver-decorated microfibrous eggshell membrane for wound healing and preventing bacterial infection^34^. In previous researches, eggshell wastes can be used to prepare different calcium carbonate purifications by many methods and then are used to synthesize advanced calcium salts such as calcium oxide, calcium hydroxide, various calcium phosphates, calcium citrate, calcium acetate, and calcium lactate^1,35–39^. Powdered eggshells have two purified calcium carbonate grades as low and high purity, which were obtained by different preparation approaches such as drying, washing, grinding, and calcining^1^. Low-purity calcium carbonate was used as animal feed, fertilizers, and heavy metal removal as described by Ok et al.^40^, whereas high-purity calcium carbonate was used in cosmetic and medical industry, as a base material for bio-ceramics, bone and dental implants, and anti-tartar toothpastes reported by many researchers^1,14,41–49^. Many researches have converted eggshells to calcium oxide (CaO)^15,50–58^ or CaO/Au nanocatalyst^59^ for use as catalyst in biodiesel productions. Other uses of eggshells are as calcium source to synthesize different calcium salts such as calcium citrate, lactate, gluconate and phosphate^1^, which are used in food additive and medicine^14^. For calcium phosphates obtained from eggshells, many researches have studied on synthesis of different tricalcium phosphate forms (α, β, or γ-Ca_3_(PO_4_)_2_) and hydroxyapatite (Ca_10_(PO_4_)_6_(OH)_2_) to use as a base material for bio-ceramics, bone and dental implants^35,36,43,46^. However, calcium phosphates produced by the commonly used synthesis routes such as precipitation, sol–gel, microwave or hydrothermal route have several drawbacks, including having the obtained materials in the form of powders. Furthermore, eggshells have still not arrived sufficient attention with regard to converting them from waste to calcium phosphate materials to be used at a large scale. Therefore, there are still a large number of eggshell wastes each day.

The main interest of proposing new routes is to produce four calcium phosphates (triple superphosphate (Ca(H_2_PO_4_)_2_·H_2_O), dicalcium phosphate dihydrate (CaHPO_4_·2H_2_O), monocalclium phosphate monohydrate ((Ca(H_2_PO_4_)_2_·H_2_O), and tricalcium phosphate (Ca_3_(PO_4_)_2_), used in fertilizer, animal feed, and food additive with enormous use in Thailand, by simple, rapid, cheap, and environmentally benign methods. The main objective of the work is to propose a new and highly competent method in terms of environmental friendliness, cost, quality, and zero-waste management. These synthesis processes offer fast reaction, easy reproducibility, high yield and without controlling various conditions to get four calcium phosphate products. The calcium raw materials and the prepared products were identified by X-ray fluorescence (XRF), X-ray diffraction (XRD), Fourier-transform infrared spectroscopy (FTIR) and Thermogravimetric analysis (TGA).

## Materials and methods

### Preparation of raw materials

**Figure 1:**
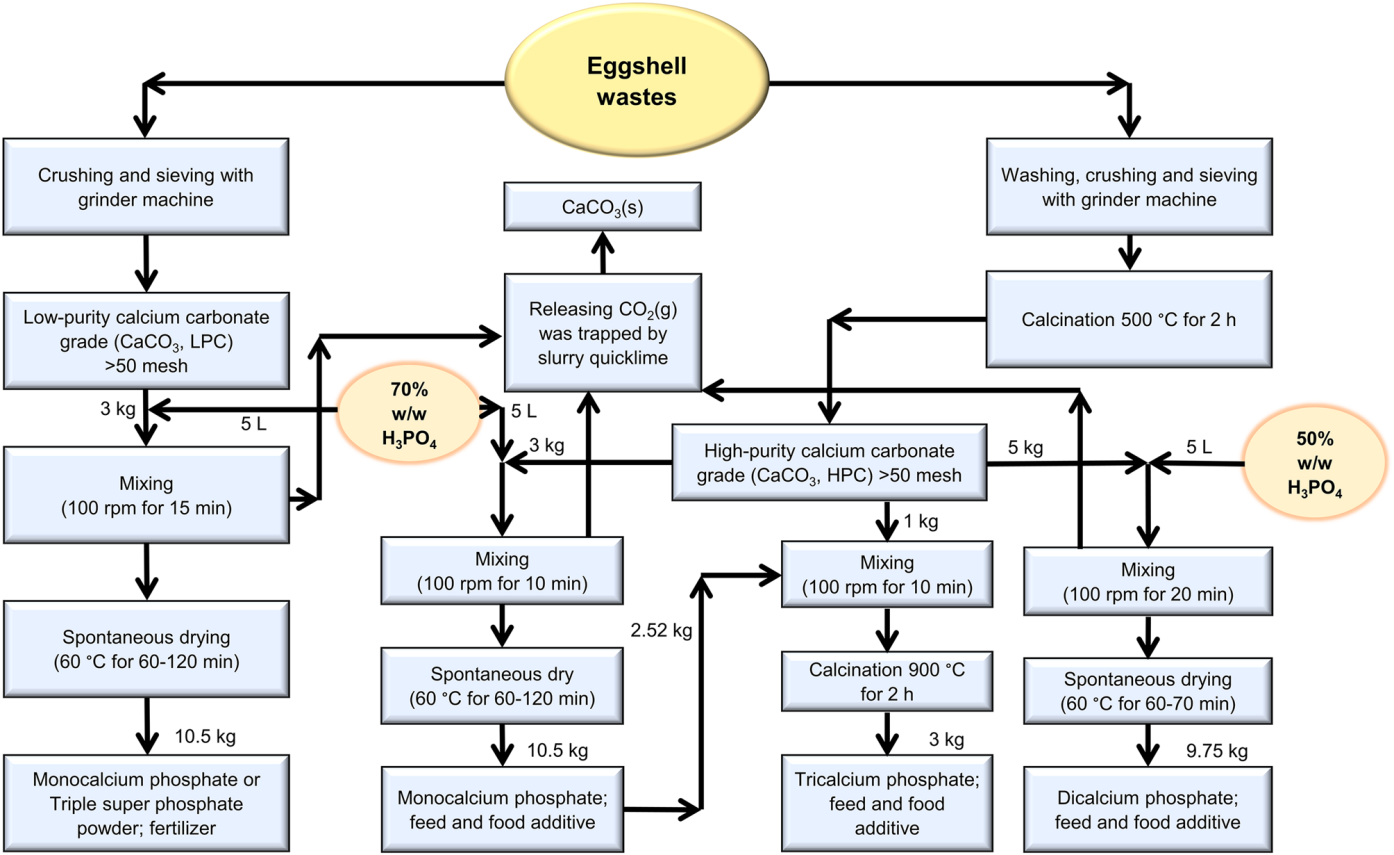
Process flowchart of low- and high-purity calcium carbonates obtained from eggshells and four calcium phosphate products.

Eggshell wastes were obtained from commercial food, bakery, and cake shop (Chachoengsao Province, Thailand), which were normally stored 2 tons a day. These wastes were turned into 2 levels of calcium carbonate purity, low- and high-purity grades, as raw materials for producing fertilizer and feed mineral, respectively. Figure 1 shows a general flowchart of the preparation process of the two calcium carbonate grades from eggshells.

Low-purity calcium carbonate (LPC) was prepared by crushing eggshells followed by sieving through a 50 mesh with grinder machine. Thus, coarse powders with purity levels of 94–97% in calcium carbonate that would be used to produce phosphate fertilizers were obtained.

High-purity calcium carbonate (HPC) was prepared by treating eggshells as follow: washed with household detergent, crushed and sieved through a 50 mesh with grinder machine and then calcined at 500 °C for 2 h with a furnace. Subsequently, coarse powders with purity levels of 97–99% in calcium carbonate that will be used to produce animal feed and food additive are obtained.

Commercial-grade phosphoric acid (85% w/w H_3_PO_4_) was used without prior purification. Two concentrations of phosphoric acid prepared by dilution with water are 50 and 70% w/w H_3_PO_4_. In a typical process, 10 kg of 85% w/w H_3_PO_4_ were added to 7 and 2.15 kg of water for preparing 50 and 70% H_3_PO_4_, respectively. The dilution reactions were strong exothermic processes which occurred at temperature range of 50–70 °C.

### Production of calcium phosphate compounds

Triple superphosphate (Ca(H_2_PO_4_)_2_·H_2_O), fertilizer containing more than 54.62% of available phosphoric acid (P_2_O_5_) was prepared as follow. At beginning procedure, 3 kg of LPC was placed in a plastic container (20 L). Then 5 L of 70% w/w H_3_PO_4_ were slowly added and followed by stirring at 100 rpm for about 15 min. The resulting reaction was strong exothermic and was aged in a plastic container for about 60–120 min to dry spontaneously and then was ground to fine powder. The white-yellow product (Ca(H_2_PO_4_)_2_·H_2_O) (Fig. 1), triple super phosphate is obtained by a simple, rapid, and low-cost production process according to Eq. (1):

1 $${\text{CaCO}}_{{3}} \left( {\text{s}} \right) \, + {\text{ 2H}}_{{3}} {\text{PO}}_{{4}} \left( {{7}0\% {\text{ w}}/{\text{w}}} \right) \, \to {\text{ Ca}}\left( {{\text{H}}_{{2}} {\text{PO}}_{{4}} } \right)_{{2}} \cdot {\text{H}}_{{2}} {\text{O}}\left( {\text{s}} \right) \, + {\text{ CO}}_{{2}} \left( {\text{g}} \right).$$

Dicalcium phosphate dihydrate (CaHPO_4_·2H_2_O), feed and food grade containing 18% of phosphorus and 24% of calcium, was produced as follows: in the starting process, 5 kg of HPC was placed in a plastic container (20 L), followed by the immediate addition of 5 L of 50% w/w H_3_PO_4_ and then stirring at 100 rpm for about 20 min. Finally, the resulting reaction which was moderately exothermic was aged in a plastic container for about 50–70 min to get dried product and then was ground to fine powders and the white product of dicalcium phosphate (CaHPO_4_·2H_2_O) is obtained (Fig. 1). This reaction is simple, quick, and cheap and is shown in Eq. (2).

2 $${\text{CaCO}}_{{3}} \left( {\text{s}} \right) \, + {\text{ H}}_{{3}} {\text{PO}}_{{4}} \left( {{5}0\% {\text{ w}}/{\text{w}}} \right) \, + {\text{ H}}_{{2}} {\text{O}}\left( {\text{l}} \right) \, \to {\text{ CaHPO}}_{{4}} \cdot {\text{2H}}_{{2}} {\text{O}}\left( {\text{s}} \right) \, + {\text{ CO}}_{{2}} \left( {\text{g}} \right).$$

Monocalcium phosphate monohydrate (Ca(H_2_PO_4_)_2_·H_2_O), feed and food grade containing 21% of phosphorus and 15% of calcium, was prepared similarly to triple superphosphate but using HPC as starting agent and the final white powder product was obtained (Fig. 1).

Tricalcium phosphate (Ca_3_(PO_4_)_2_) (Fig. 1), food grade containing 44% of phosphorus and 38% of calcium, was synthesized as follows: in a typical route, 2.52 kg of monocalcium phosphate monohydrate (Ca(H_2_PO_4_)_2_·H_2_O) and 1.0 kg of HPC were thoroughly mixed by ball milling. Then the resulting mixture was transferred into a ceramic container and then was calcined at 900 °C for 2 h in a furnace. The calcined product was obtained as white powder corresponding to Eq. (3).

3 $${\text{2CaCO}}_{{3}} \left( {\text{s}} \right) \, + {\text{ Ca}}\left( {{\text{H}}_{{2}} {\text{PO}}_{{4}} } \right)_{{2}} \cdot {\text{H}}_{{2}} {\text{O}}\left( {\text{s}} \right) \, \to {\text{ Ca}}_{{3}} \left( {{\text{PO}}_{{4}} } \right)_{{2}} \left( {\text{s}} \right) \, + {\text{ 2CO}}_{{2}} \left( {\text{g}} \right) \, + {\text{ 3H}}_{{2}} {\text{O}}\left( {\text{g}} \right)\,{\text{at}}\,{9}00\,^\circ {\text{C}}{.}$$

### Characterization

The starting materials and as-prepared products were obtained and identified. Four analyses were carried out in this study: X-ray Fluorescence (XRF, Bruker S4 Pioneer), X-Ray Diffraction (XRD, Bruker D8 Advance), Fourier-Transform Infrared Spectrophotometer (FTIR, Perkin Elmer Spectrum GX) and Thermogravimetric Analyzer (TGA, Perkin Elmer Pyris I). The crystal structures of samples were estimated using XRD in 2θ range of 1°–60° with a step size of 0.05 and a step time of 1 s. The room temperature FTIR spectra were recorded in the range of 4000–400 cm^−1^ with eight scans on a Perkin-Elmer Spectrum GX spectrometer with the resolution of 4 cm^−1^. Thermal analysis was used to characterize water content in the as-prepared samples as dicalcium phosphate dihydrate, triple superphosphate, and monocalcium phosphate monohydrate.

## Results and discussion

### Production results

Percentage yields of productions were found to be 98, 97, 98 and 96 for triple superphosphate, dicalcium phosphate dihydrate, monocalcium phosphate monohydrate, and tri calcium phosphate, respectively. Chemical contents and purity of starting agents and all as-prepared products are checked by XRF method and tabulated in Table 1. These results confirm chemical formula to be CaCO_3_ (Ca:O ratio of 1.10:3.31 for LPC and 1.09:3.38 for HPC), CaHPO_4_·2H_2_O (Ca:P ratio of 1.00:1.02), Ca(H_2_PO_4_)_2_·H_2_O (Ca:P ratio of 1.00:2.02 for triple superphosphate and 1.00:2.05 for monocalcium phosphate dihydrate) and Ca_3_(PO_4_)_2_ (Ca:P ratio of 1.47:1.00), which are well consistent with theoretical information. These obtained data are important for their applications such as fertilizer, feed and food because the standard of materials is indicated.

**Table 1:** Compositions (± 0.02%) of starting agents (CaCO_3_ obtained from eggshells) and as determined by X-ray fluorescence (XRF). Percentage values less than 0.02% (limit of detection) were omitted for clarity.

Samples	Percentage of metal content (%)									Product purity	
	CaO	P_2_O_5_	LOI	MgO	SiO_2_	K_2_O	CuO	SO_3_	Fe_2_O_3_	Formula	%
Low-purity calcium carbonate (LPC)	53.25	0.74	41.48	1.65	0.56	0.41	0.28	0.93	0.71	CaCO_3_	95.09
High-purity calcium carbonate (HPC)	55.28	–	40.82	1.47	0.28	0.36	0.21	1.13	0.46	CaCO_3_	98.71
Triple superphosphate (fertilizer grade)	21.32	54.62	18.18	2.08	0.92	0.77	0.31	0.88	0.91	Ca(H_2_PO_4_)_2_·H_2_O	96.66
Dicalcium phosphate dehydrate (feed animal and food grade)	33.04	42.83	20.91	0.32	1.37	0.8	0.15	0.28	0.31	CaHPO_4_·2H_2_O	99.54
Monocalcium phosphate monohydrate (feed animal and food grade)	22.4	55.88	18.7	0.52	0.58	1.3	0.11	0.28	0.23	Ca(H_2_PO_4_)_2_·H_2_O	99.63
Tricalcium phosphate (feed animal and food grade)	53.21	45.05	< 0.1	0.62	0.11	0.29	0.09	0.38	0.2	Ca_3_(PO_4_)_2_	98.26

### Characterization results

**Figure 2:**
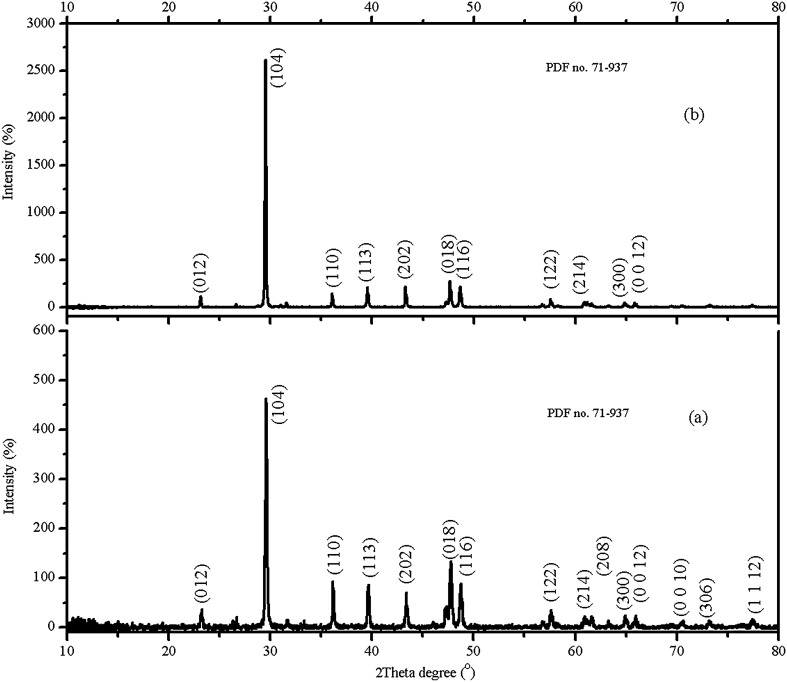
X-ray diffraction patterns of low-purity (a) and high-purity (b) calcium carbonates obtained from powdered eggshell samples.

**Figure 3:**
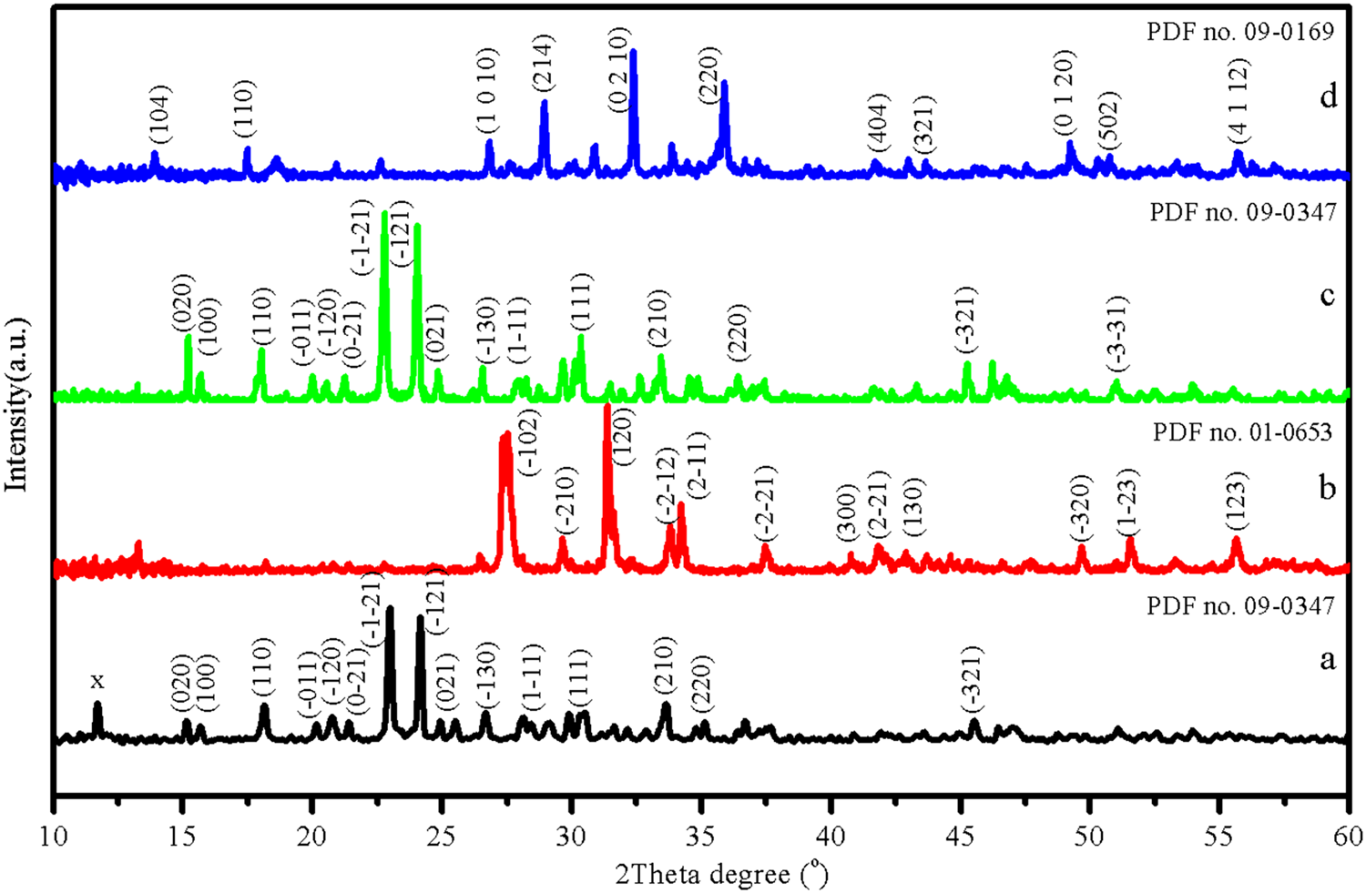
X-ray diffraction patterns of triple superphosphate (a), dicalcium phosphate dihydrate (b), monocalcium phosphate monohydrate (c), and tricalcium phosphate (d).

The XRD patterns from powder samples of the starting agents and the as-prepared products are shown in Figs. 2 and 3, respectively. Based on XRD spectra, the starting agents (LPC and HPC) contained calcite (CaCO_3_, PDF no. 71-937)^3^. However, the peak intensities of calcite in LPC sample are lower than those of HPC sample because of the impurity effect. The XRD pattern of as- prepared dicalcium phosphate dihydrate (CaHPO_4_·2H_2_O) (Fig. 3b) belongs to brushite (CaHPO_4_·2H_2_O, PDF no. 01-0653)^41^. The XRD patterns of as-prepared triple superphosphate (Fig. 3a) and monocalcium phosphate monohydrate (Fig. 3c) are very similar but lower and higher peak intensities are observed because the products were obtained from different purities of calcium carbonate grades^36^. The lower peak intensities were observed in the XRD pattern of as-prepared triple superphosphate due to the LPC was used as the starting agent. For as-prepared tricalcium phosphate, diffraction lines attribute mainly to beta-tricalcium phosphate (Ca_3_(PO_4_)_2_, PDF no. 70-2065)^42^. Based on the XRD observation, it can be concluded that no impurity appeared in as-prepared HPC, dicalcium phosphate dihydrate, monocalcium phosphate monohydrate, and tricalcium phosphate powders, and some impurities appeared in as-prepared LPC and triple superphosphate samples.

**Figure 4:**
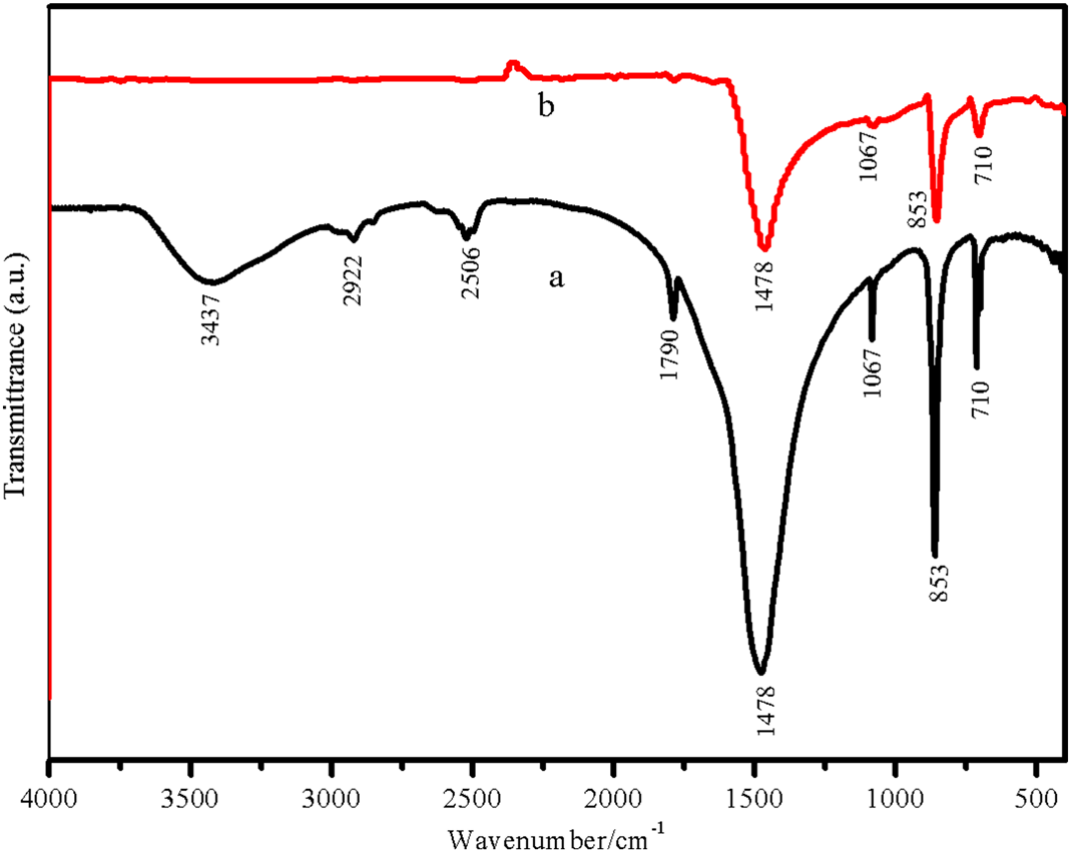
FTIR spectra of low-purity (a) and high-purity (b) calcium carbonates obtained from powdered eggshell samples.

**Figure 5:**
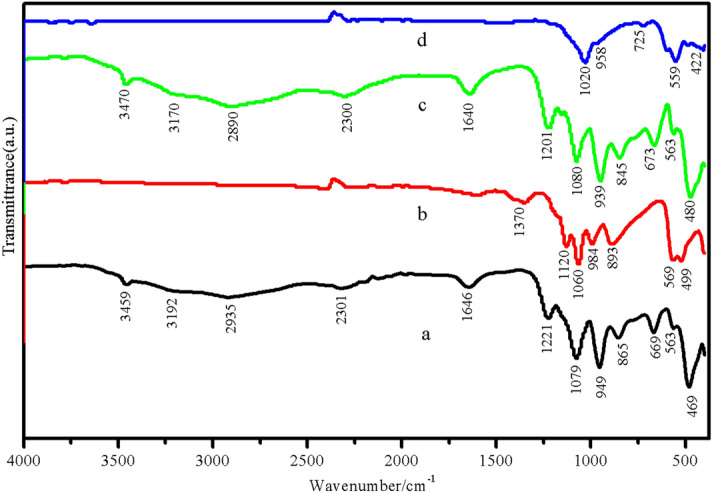
FTIR spectra of triple superphosphate (a), dicalcium phosphate dihydrate (b), monocalcium phosphate monohydrate (c), and tricalcium phosphate (d).

Figures 4 and 5 show FTIR spectra of the starting agents (LPC and HPC) and the as-prepared products. The FTIR spectra of as-prepared LPC (Fig. 4a) and HPC (Fig. 4b) are very similar and assigned according to vibrational modes of CO_3_^2−^ and Ca–O, which are block units of calcium carbonate (CaCO_3_). The appeared prominent absorption peak of CO_3_^2−^ at 1478 cm^−1^ corresponds to the asymmetric stretching mode of C–O bond. The appeared vibrational peaks at 1067, 853, and 710 cm^−1^ are assigned to the symmetric stretching mode of C–O bond, out of plane and in plane bending of CO_3_^2−^ anion, respectively^3^. The FTIR spectra of triple superphosphate (Fig. 5a) and monocalcium phosphate monohydrate (Fig. 5b) are very similar and assigned based on the fundamental vibration of Ca(H_2_PO_4_)_2_·H_2_O. For triple superphosphate (Fig. 5a), the strong bands in the region of 1150–950 and 450–600 cm^−1^ are attributed to the P–O stretching vibrations and the bending OPO vibrations, respectively. The couple bands at 1221 and 865 cm^−1^ are assigned to the in-plane P–O–H bending (*A*_2_), and the out of plane bending (*A*_1_) vibration, respectively. A weak band occurred at approximately 669 cm^−1^ is assigned to rocking mode involving water molecules. In the O–H stretching mode region of the H_2_PO_4_^−^, which are characteristic for this compound, they appear as the A, B, C trio bands at about 3100–3200 cm^−1^ (A band), 2300–2400 cm^−1^ (B band), and 1600–1800 cm^−1^ (C band). The vibrational bands of water molecule appear in three modes at 1646 cm^−1^ (ν_2_), 3192 cm^−1^ (ν_1_), and 3459 cm^−1^ (ν_3_) and are assigned to bending, asymmetric stretching (O–H), and antisymmetric stretching (O–H) modes, respectively^60^. Vibrational modes of as-prepared dicalcium phosphate dihydrate (Fig. 5b) are assigned according to the units of HPO_4_^2−^ ion, H_2_O, and CaO. The typical intense bands at about 1370 cm^−1^ is due to the in-plane P–O–H bending, while the out of plane bending vibration is observed at about 893 cm^−1^. The strong bands at about 1120, 1060, and 984 cm^−1^ are assigned to PO_3_ asymmetric stretching modes. The weak and broader band at about 569 cm^−1^ is corresponding to PO_4_ and P–OH bending modes. The band at 499 cm^−1^ is assigned to PO_3_ symmetric bending or Ca–O stretching^61^. The FTIR spectrum shown in Fig. 5c is very similar to the FTIR spectrum as shown in Fig. 5a because of fundamental vibrational block units (H_2_PO_4_^−^, CaO, and H_2_O) that come from the same formula (Ca(H_2_PO_4_)_2_·H_2_O), resulting in closely similar vibrational frequencies^60^. Finally, the PO_4_^3−^ and CaO subunits of Ca_3_(PO_4_)_2_ structure are assigned (Fig. 5d). Vibrational bands of PO_4_^3−^ anion of the product are observed in the regions of 450–610 and 900–1000 cm^−1^. These bands are assigned to the ν_4_(PO_4_^3–^) and ν_3_(PO_4_^3–^) vibrations, respectively. Additionally, the observation of a strong ν_s_(POP) bands (725 cm^−1^) is known to be the most striking feature of polyphosphate spectrum. The FTIR results are consistent with X-ray diffraction data.

**Figure 6:**
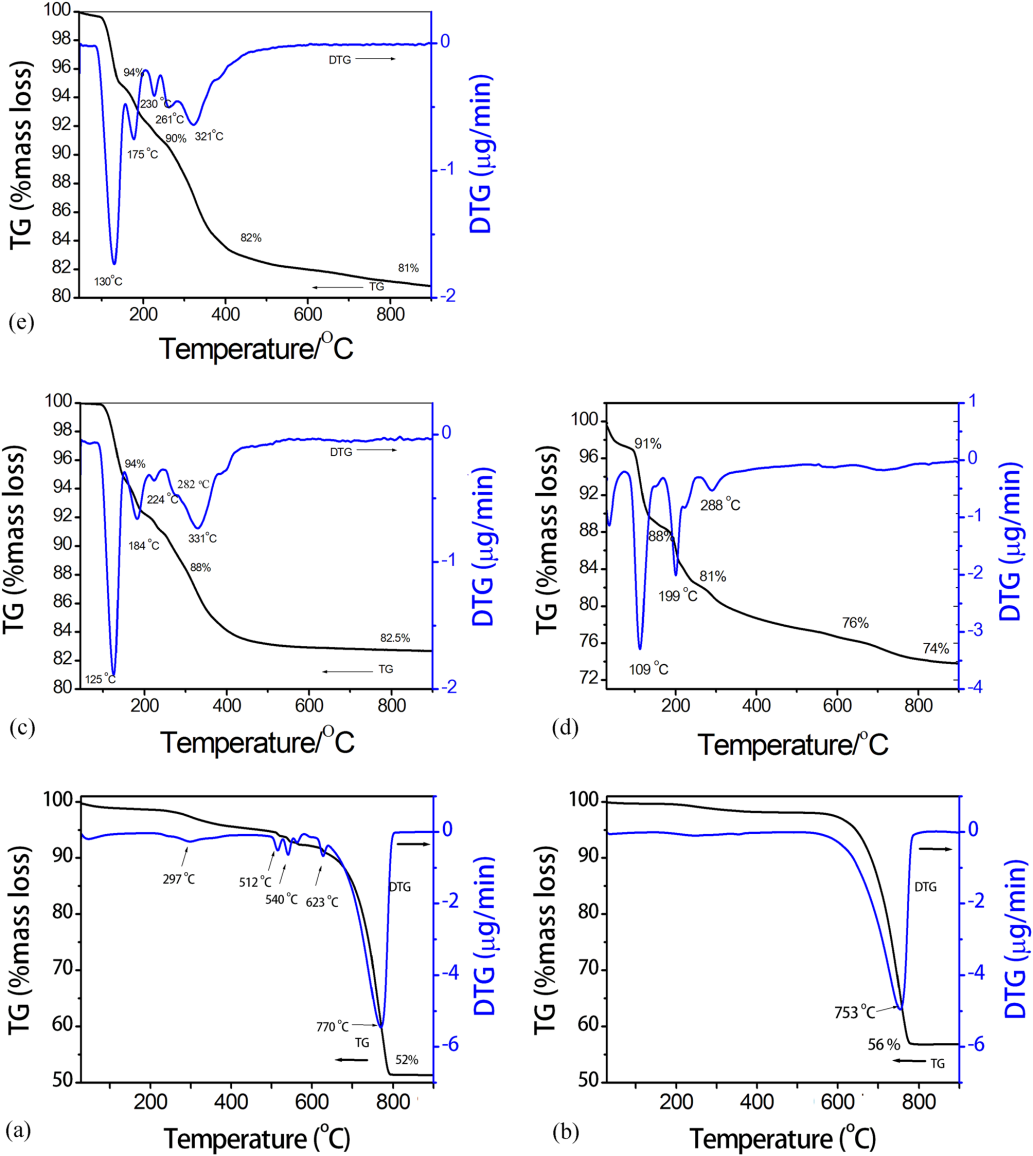
TG/DTG curves of LPC (**a**), HPC (**b**), triple superphosphate (**c**), dicalcium phosphate dihydrate (**d**), and monocalcium phosphate monohydrate (**f**).

Thermal behaviors of the starting agents (LPC and HPC) and only three as-prepared products (triple superphosphate, dicalcium phosphate dihydrate and monocalcium phosphate monohydrate) investigated by TG/DTG technique are shown in Fig. 6. Tricalcium phosphate is an anhydrous compound, which is not necessary to analyze thermal decomposition. TG/DTG curves of the LPC (Fig. 6a) and HPC (Fig. 6b) samples are quite similar. TG curves of each sample show the mass loss in the range of 600–800 °C, which corresponds to a strong single peak of DTG curves at 770 and 753 °C for the LPC and HPC samples, respectively. Many DTG peaks observed at 297, 512, 540 and 623 °C indicate clearly impurity of the LPC sample. The quantities of mass loss (remaining mass) are found to be 48% (52%) and 44% (56%) for LPC and HPC samples, respectively. The thermal behavior obtained confirms that the LPC sample has a lower purity than the HPC sample. However, the thermal results obtained were well consistent with those of the reference and theory information of CaCO^48,49^. The thermal results indicate that LPC and HPC powders can be transformed to CaO by calcination at above 800 °C, which may be useful for the production of this compound used in specific applications, revealed by:

4 $${\text{CaCO}}_{{3}} \to {\text{CaO}} + {\text{CO}}_{{2}} \left( {\text{g}} \right).$$

TG curves of triple superphosphate and monocalcium phosphate monohydrate are observed in the range of 30–900 °C. The mass remains occurred at above 700 °C, which are found to be 82.50 and 81.00% for triple superphosphate (Fig. 6c) and monocalcium phosphate monohydrate(Fig. 6e), respectively. The mass losses found to be around 18% (2.57H_2_O) for each sample are related to the loss of three water molecules in Ca(H_2_PO_4_)_2_·H_2_O structure. The masses of loss and remain observed from each sample are close to that of theoretical values at 21 (3.0H_2_O) and 79%, respectively^53^. The relative with TG data, five DTG peaks of both samples are observed the same but peak positions are slightly different. Fiver DTG peaks of triple superphosphate and monocalcium phosphate monohydrate are observed at 125, 184, 224, 282, 331 °C and 130, 175, 230, 261, 321 °C, respectively. Mechanism reactions of thermal transformation for both compounds could be:

5 $${\text{Ca}}\left( {{\text{H}}_{{2}} {\text{PO}}_{{4}} } \right)_{{2}} \cdot {\text{nH}}_{{2}} {\text{O}}\left( {\text{s}} \right) \to {\text{Ca}}\left( {{\text{H}}_{{2}} {\text{PO}}_{{4}} } \right)_{{2}} \cdot {\text{mH}}_{{2}} {\text{O}}\left( {\text{s}} \right) \, + {\text{oH}}_{{2}} {\text{O}}\left( {\text{g}} \right),$$

6 $${\text{Ca}}\left( {{\text{H}}_{{2}} {\text{PO}}_{{4}} } \right)_{{2}} \cdot {\text{mH}}_{{2}} {\text{O}}\left( {\text{s}} \right) \to {\text{Ca}}\left( {{\text{H}}_{{2}} {\text{PO}}_{{4}} } \right)_{{2}} \cdot {\text{pH}}_{{2}} {\text{O}}\left( {\text{s}} \right) \, + {\text{qH}}_{{2}} {\text{O}}\left( {\text{g}} \right),$$

7 $${\text{Ca}}\left( {{\text{H}}_{{2}} {\text{PO}}_{{4}} } \right)_{{2}} \cdot {\text{pH}}_{{2}} {\text{O}}\left( {\text{s}} \right) \to {\text{Ca}}\left( {{\text{H}}_{{2}} {\text{PO}}_{{4}} } \right)_{{2}} \left( {\text{s}} \right) + {\text{pH}}_{{2}} {\text{O}}\left( {\text{g}} \right),$$

8 $${\text{Ca}}\left( {{\text{H}}_{{2}} {\text{PO}}_{{4}} } \right)_{{2}} \left( {\text{s}} \right) \to {\text{CaH}}_{{2}} {\text{P}}_{{2}} {\text{O}}_{{7}} \left( {\text{s}} \right) + {\text{H}}_{{2}} {\text{O}}\left( {\text{g}} \right),$$

9 $${\text{CaH}}_{{2}} {\text{P}}_{{2}} {\text{O}}_{{7}} \left( {\text{s}} \right) \to {\text{Ca}}\left( {{\text{PO}}_{{3}} } \right)_{{2}} \left( {\text{s}} \right) + {\text{H}}_{{2}} {\text{O}}\left( {\text{g}} \right),$$

where n = o + p + q ≅ 1.

A large amount of intermediate compounds, such as Ca(H_2_PO_4_)_2_·mH_2_O, Ca(H_2_PO_4_)_2_·pH_2_O, acid polyphosphate Ca(H_2_PO_4_)_2_, acid condensed phosphate CaH_2_P_2_O_7_ and mixtures of intermediate have been registered. Calcium polyphosphate (Ca(PO_3_)_2_) was found to be the product of the thermal decomposition of both prepared compounds over 800 °C as revealed by the TG curve. This result may be not in agreement with literature^53^, which reported in the range of 500–700 °C of this transition phase. TG curve of dicalcium phosphate dihydrate (CaHPO_4_·2H_2_O) (Fig. 6d) show the mass loss in the range of 100–800 °C. The total mass loss is 26%, which is closely to the theoretical value (26.16%, 2.5H_2_O). The relative with TG data, three intense DTG peaks are observed at 109, 199 and 288 °C, respectively. The formally reactions of thermal transformations could be:

10 $${\text{CaHPO}}_{{4}} \cdot {\text{2H}}_{{2}} {\text{O}}\left( {\text{s}} \right) \to {\text{CaHPO}}_{{4}} \cdot {\text{xH}}_{{2}} {\text{O}}\left( {\text{s}} \right) + {\text{ yH}}_{{2}} {\text{O}}\left( {\text{g}} \right),$$

11 $${\text{CaHPO}}_{{4}} \cdot {\text{xH}}_{{2}} {\text{O}}\left( {\text{s}} \right) \, \left( {\text{s}} \right) \to {\text{CaHPO}}_{{4}} \left( {\text{s}} \right) + {\text{ xH}}_{{2}} {\text{O}}\left( {\text{g}} \right),$$

12 $${\text{CaHPO}}_{{4}} \left( {\text{s}} \right) \to {1}/{\text{2Ca}}_{{2}} {\text{P}}_{{2}} {\text{O}}_{{7}} \left( {\text{s}} \right) + {1}/{\text{2H}}_{{2}} {\text{O}}\left( {\text{g}} \right).$$

The intermediate compounds including CaHPO_4_·xH_2_O, CaHPO_4_(s) and mixtures of intermediate have been registered. Calcium pyrophosphate (Ca_2_P_2_O_7_) was found to be its final decomposition product 800 °C as revealed by the TG curve. This result may be not in agreement with literature^2,4,33,35^, which reported lower temperature (< 700 °C) of this transition phase. Thermal results of each compound indicate clearly that the property dependent on various conditions (preparation technique, synthesis condition, raw material, and so on).

The novelty of this work is the production methods that are simple, rapid, cost-saving and environmentally benign for the obtained calcium carbonate (raw material) and four calcium phosphate compounds, shown in Fig. 1. The eggshell wastes were accumulated about 60 tons/month from only one bakery shop in Chachoengsao Province, Thailand. Transportation of these wastes (60 tons) in the distance of 500–800 km is about 1242 USD, which indicates the logistic cost of eggshell no more than 0.021 USD/kg. The eggshells were prepared by two ways to get particle size of 50 mesh. In the first method, they were crushed only to become low-purity calcium carbonate grade, which was in the cost about 0.0031 USD/kg. In the second method, they were washed with detergent and crushed to become high calcium carbonate grade, which had the cost of about 0.009 baht/kg. The ways of the prepared eggshells turn into low- or high-purity calcium carbonates in this work are similar with previous reports^1,3^. Calcium carbonate (CaCO_3_) has three crystal forms as calcite, aragonite, and vaterite and is occurred from two sources: living and non-living sources. Non-living source is found in limestone mineral such as calcite (CaCO_3_) and dolomite (Ca_x_Mg_y_CO_3_, x + y = 1), which may have impurities of other heavy metals such as lead (Pb), cadmium (Cd), arsenic (As), etc. and has limited quantity, which may run out in the future. The living source is found as the main component of pearls and the shells of marine organisms, snails, and eggs. It happens every day and is collected as large volume of wastes and without heavy toxic metal according to theory of natural selection. The low- or high-purity calcium carbonates prepared from eggshells reported in this work were investigated by XRF, XRD and FTIR techniques for confirmation of nontoxic impurities, phases, and structures. Two calcium carbonate grades obtained in this work were used as calcium sources to react with specific phosphoric acid concentration at very short time (< 30 min) and spontaneously dry solid products were obtained as superphosphate for phosphorus fertilizer (56% P_2_O_5_), monocalcium phosphate, dicalcium phosphate, and tricalcium phosphate for feed animal and food additive. In the production of some calcium phosphates, carbon dioxide (CO_2_) gas was evolved in the process and was tapped by quicklime to turn into calcium carbonate, which is a similar method to previous reports^62,63^. The production methods of four calcium phosphates reported in this work were simple, rapid, cheap, and safe methods when compared with methods reported in literatures^2,4,25,36,41^ and the resulting costs were 16, 11, 18 and 24 baht/kg for triple superphosphate, dicalcium phosphate dihydrate, monocalcium phosphate monohydrate, and tricalcium phosphate, respectively. The characterizations of four calcium phosphates were performed using XRF, XRD and FTIR techniques. Impurities, phases and structures were used to confirm that the obtained products were “Green Products” (no metal toxic impurities and according to standard material product) to replace the same products in Thailand markets. All obtained based on this work lead to transforming or recycling of eggshells to four calcium phosphates with lower or near prices and “green products” compared with the same products in the markets. Therefore, eggshell wastes from anywhere can be used to prepare calcium phosphates by methods reported in this work, which will be alternatives to remove these wastes from environmental problem in the future. The authors suggest that pilot projects are required to evaluate their implementation to a commercial scale by using these cleaner production processes to convert waste into industrially viable resources in the future.

## Conclusions

Waste eggshells represented one of the most urging problems of agricultural and food industries as well as communities, due to their enormous amount that must be disposed of in some way nowadays. This research described an alternative way to recycle wastes as added-value products to solve eggshell problem of industries and communities. The as-prepared raw materials (low- and high-purity calcium carbonate (LPC and HPC) and products (triple superphosphate, dicalcium phosphate dihydrate, monocalcium phosphate monohydrate, and tricalcium phosphate) were produced by simple, rapid, cheap, and environmentally benign method. Identification of structural phase and impurity of all as-prepared samples was confirmed by XRF, TGA, XRD and FTIR techniques. The findings indicate that all as-prepared samples can be used in industries of fertilizer, animal feed and food additive, which require massive amounts. Therefore, the study represents alternative ways of the environmental benefits resulting from possible transformation of eggshells to value-added calcium compounds needed for wide uses in many industries. If the eggshells are recycled as described above, then a future of these wastes will be used to produce value-added products, which take care of the environment according to the way of zero waste and corresponding to sustainable development.
